# Probiotic management and inflammatory factors as a novel treatment in cirrhosis: A systematic review and meta-analysis

**DOI:** 10.1515/biol-2022-0741

**Published:** 2023-10-17

**Authors:** Qinglan Xia, Yumeng Lei, Jiadun Wang, Qiang Wang

**Affiliations:** Institute of Infection, Immunology and Tumor Microenvironment, Hubei Province Key Laboratory of Occupational Hazard Identification and Control, School of Medicine, Wuhan University of Science and Technology, Wuhan 430065, China; Asia General Hospital Affiliated to Wuhan University of Science and Technology, Wuhan 430056, China

**Keywords:** Probiotic, gut microbiota, gut–liver axis, inflammation factors, cirrhosis

## Abstract

The interaction between intestinal microecological dysregulation, altered inflammatory factors, and cirrhosis is unclear. The aim of this systematic review and meta-analysis was to synthesize the results of previous studies to assess the efficacy of probiotics in the treatment of cirrhosis and their effect on inflammatory factors, as well as to explore the relationship between gut microecological dysregulation and liver disease to gain a deeper understanding of this interaction. Up to December 2022, eligible studies were identified by searching the following databases: National Knowledge Infrastructure (CNKI), Wanfang Data, Web of Science, PubMed, Embase, Medline, and the Cochrane Library. Statistical analysis was performed using software RevMan Version 5.4. A total of 33 eligible randomized controlled trials were included in the study, and data on probiotic strains, duration of intervention, measures in the control group, and outcomes were extracted and evaluated. Compared to the control group, the experimental group had significant improvements in overall efficacy. The results of the meta-analysis revealed that probiotic use significantly decreased biochemical parameters for liver function, including aspartate transaminase, alanine aminotransferase, and total bilirubin. Similar result was obtained in interleukin-6, tumor necrosis factor-α, and endotoxin. However, probiotic intervention did not significantly affect interleukin-2 and interleukin-10. The current meta-analysis illustrates that probiotic supplementation reduces inflammatory markers and biochemical parameters for liver function in patients with cirrhosis, suggesting that probiotic management may be a novel treatment for cirrhosis. Furthermore, the interaction of the gut microbiota, associated metabolites, and inflammation factors with cirrhosis may provide a promising therapeutic target for the pharmacological and clinical treatment of cirrhosis.

## 1 Introduction

Liver cirrhosis is widely prevalent and is associated with high morbidity and mortality. With roughly one million annual deaths attributable to the disease [1], it is the 11th most common cause of death worldwide [2]. Cirrhosis is the seventh leading cause of disability-associated life years in the 50–74 age group, the 12th top cause in people aged 25–49 years, and the 15th highest cause in all ages [3]. The most common etiologies of cirrhosis globally are non-alcoholic fatty liver disease, alcoholic liver disease, and chronic hepatitis B and chronic hepatitis C. Regardless of the cause, the complications of cirrhosis are similar, including hepatic encephalopathy (HE) [4]. Cirrhosis has put a strain on global public health; hence, efficient and safe treatment options are urgently needed.

The World Health Organization updated the definition of a probiotic: “live microorganisms which when administered in adequate amounts confer a health benefit on the host” [5]. Probiotics are part of the human microbial community, most of which are beneficial bacteria naturally present in the body. Different strains can be isolated from different parts of the body, including the gastrointestinal tract, mouth, skin, and uterus. Other bacterial genera such as *Streptococcus*, *Enterococcus* , *Yeast* , and *Bacillus* can also have probiotic properties [6]. While most parts of the body are colonized by microorganisms, the highest number of microorganisms are found in the gut. The most studied species include *Lactobacillus* and *Bifidobacterium* [7]. *Lactobacillus* and *Bifidobacterium* produce lactic acid, acetic acid, and propionic acid, which lower intestinal pH and inhibit the growth of various pathogenic bacteria, thereby reestablishing the balance of intestinal flora [8,9]. Probiotics have been well investigated in relation to numerous gastrointestinal disorders, leading to a growing consensus that probiotics, the gut microbiota, and both health and disease in the gut are strongly correlated [10,11].

Probiotics are one of the most commonly used food supplements in the health-care industry worldwide [12]. Since approximately 70% of the immune system is located in the intestine [13], it can be further concluded that probiotics typically provide two general benefits: supporting a healthy digestive tract and a healthy immune system. This conclusion was based on a great deal of reliable research, including clinical trials, meta-analyses as well as reviews, suggesting that probiotics can be expected to have effects on diverse disease conditions such as gastrointestinal disease [14,15,16,17], autoimmune disease [18,19,20], diabetes [21,22,23,24], hypertension and hyperlipidemia [25,26], as well as several kinds of liver disease [27,28,29,30]. Probiotics induce their effects primarily due to their role in immune system regulation and anti-inflammatory response [31]. Thus, probiotic administration seems to have a great potential in terms of health, and further research should be conducted. More and more attention has been paid to the health benefits of probiotics, which we believe to be potentially crucial interventions for improving health and well-being.

Probiotic research has rapidly expanded, inspiring scientists to create a wide range of functionally formulated products with validated health advantages. Probiotics, prebiotics, vitamins, and minerals are among the functional food ingredients that can be found in a variety of meals and beverages, including fermented milks, yogurts, and sports drinks [32,33,34]. Customers can benefit nutritionally from probiotics and fermented food, which also strengthen the immune system to fend off infections [35,36]. The demand for probiotic food is expanding quickly on a global scale. Today, 60 to 70 percent of the market for functional food is made up of probiotic products [5,37]. The effectiveness of probiotic is species-, dose-, and disease-specific, and with the selection of appropriate strains, products, and dosages, probiotics may be beneficial in achieving positive effects in different disease areas [38,39]. Probiotics are useful in treating irritable bowel syndrome and ulcerative colitis, according to solid research [40,41]. Importantly, there is strong information about the advantages of probiotics in the prevention and treatment of diarrhea brought on by antibiotic use and *Clostridium difficile* [42]. Notably, two probiotic medications, Lactasin and Pepcid (*Bifidobacterium* triplex capsules), have been recommended as typical therapeutic drugs for people with cirrhosis in China, which are suitable for patients who experience abdominal pain, bloating, and diarrhea.

The term “gut–liver axis” was first proposed in 1998 by Marshall [43]. The gut–liver axis refers to the bidirectional relationship between the gut and its microbiota, allowing for the direct transport of intestinal microbes or associated metabolites to the liver via the biliary tract, portal vein, and systemic circulation, where they affect numerous liver functions, as well as a liver-to-gut feedback pathway in which it controls metabolic function and influences microbiota homeostasis and intestinal barrier integrity [44]. Imbalance of the intestinal microbiota will lead to endotoxemia and inflammation, and in the portal circulation, these effects may directly trigger or exacerbate pre-existing liver damage through harmful metabolic consequences, leading step-by-step to hepatic steatosis, chronic hepatitis, fibrosis, and cirrhosis, and the progression of cirrhosis to hepatocellular carcinoma [45]. At present, it is generally believed that inflammation is also the major factor leading to the occurrence and development of liver injury. A great deal of studies on the gut–liver axis suggest that the gut microbiota and associated metabolites may initiate a cytokine cascade that contributes to the maintenance of a poor immune response [45,46]. The adverse inflammatory responses in the liver will release a substantial amount of various serum inflammatory markers, including tumor necrosis factor (TNF), interleukins (ILs), and other general immunity markers. These inflammatory factors may be potential targets for the clinical treatment and prognosis of cirrhosis.

Guidelines advocate lactulose to lower hospitalization and fatality rates in HE in addition to the most widely used microecological treatments in clinical practice today [47,48]. Lactulose can induce the release of immunoglobulin A (IgA) from the intestinal mucosal epithelial zone as a first-line treatment for HE. IgA promotes the growth of *Bifidobacterium* and *Lactobacillus*, reduces inflammatory factors, and enhances liver function. IgA can effectively resist microbial invasion by bacteria, viruses, fungi, and other microorganisms, which helps to improve biological resistance to diseases [49]. Adverse reactions such as diarrhea, nausea, and vomiting may occur after taking lactulose oral solution, whereas probiotics have fewer adverse reactions than lactulose because they are naturally present in the body itself.

For the treatment of liver diseases, traditional Chinese medicine (TCM) has a strong theoretical foundation and a wealth of practical experience. TCM has two functions in immunological regulation: immune activation and immune suppression. These functions include the stimulation of immune cells, immune organs, and cytokine synthesis as well as the inhibition of inflammation [50]. TCM has been linked to intestinal flora, cirrhosis, and liver disease in long-term theoretical and practical investigations. This association has a special therapeutic function in the development of CLD [51,52]. Dahuang, Huangqi, and Muxiang are a few specific TCM formulae that have been shown to have anti-hepatic fibrosis benefits in addition to improving endotoxemia, lowering serum inflammatory cytokine levels, and correcting intestinal microecology in cirrhotic patients [53]. Consequently, TCM is a promising therapeutic agent for liver illness, but because of its numerous medicinal components and broad range of targets, treatment is more ambiguous and challenging [53]. The modification of intestinal homeostasis, anticirrhotic activity, and efficacy against inflammatory factors have all been demonstrated for probiotics, lactulose, and herbs. Probiotics have the ability to directly colonize the digestive tract, whereas lactulose and certain specific TCM indirectly encourage the multiplication of beneficial bacteria through medicinal components, regulating the human body.

Several studies have reported the effects of different probiotic strains on host immune factors: Toll-like receptor 2 mediated by *Lactobacillus lactis* stimulates TNF-α secretion [54], IL-10, and TNF-α secretion stimulated by *Bifidobacterium lognum* [55]; sortase-dependent pili of *Bifidobacterium bifidum* evoke the TNF-α response [56]; cell-surface polysaccharides in *Lactobacillus longum* modulate pro-inflammatory cytokine and T helper cell 17 (TH17) responses [57]; and immunostimulatory cell surface appendages (known as SpaCBA) in *Lactobacillus rhamnosus* mediate the regulation of TNF-α, IL-6, IL-10, and IL-12 [58]. Various cellular components of gut barrier (GB) (e.g., TNF, interleukins, AMP, defensins, lysozyme) have specific roles in regulating intestinal homeostasis [59,60,61,62]. Ecological imbalance and GB dysfunction are directly related to the development, progression, and appearance of clinical events linked to hepatic and portal hypertension [63]. As a result of their direct interactions with immune cells, probiotics play a significant part in preserving the body’s immunological homeostasis [64]. Probiotics can enhance the body’s immunity by increasing natural killer cell activity and stimulating phagocytosis, inhibiting pro-inflammatory cytokines, and increasing immunoglobulin concentrations, and they have a positive effect on the expression of immune-related genes, inflammatory pathway activity, and the levels of immune markers, including intestinal epithelial cell nuclear factor kappa B, mitogen-activated protein kinase, CRP, IL-6, IL-8, TNF-α, IL-1β, and IFN-γ [65,66]. Clinical outcomes and survival in cirrhotic patients have been demonstrated to be enhanced by microbial manipulation using pre-/probiotics and fecal microbiota transplantation (FMT) [63].

The immunomodulatory activity of different probiotic strains has been well documented in *in vivo*, *in vitro*, and animal studies, and specific metabolites such as polyunsaturated fatty acids, polyphenols, flavonoids, and vitamins in probiotic bacteria help to stimulate the growth and survival of beneficial gut microbes [31,67,68]. Specific molecules produced by probiotics have also been shown to be active in their immunomodulatory and anti-inflammatory pleiotropic effects. In addition to inducing the generation of anti-inflammatory cytokines, several postbiotic fractions (supernatants, cell wall fragments) recovered from *Bacillus coagulans* cultures also encouraged adjuvant T(Th)2-dependent immune responses [69]. Additionally, multiple *in vitro* studies have demonstrated that supernatants from *Bifidobacterium* short-term cultures promote DC survival and maturation, which in turn boosts IL-10 secretion and reduces TNF-α secretion [70].

However, there are few meta-analyses on the effect of probiotic interventions on cirrhotic patients in China and abroad, and most of them focus only on the changes in liver function. To the best of our knowledge, no meta-analysis has simultaneously evaluated the effects of probiotic interventions on liver function indicators and a full range of cytokines in patients with cirrhosis and the interaction between changes in gut microbiota and inflammation markers in cirrhosis remains elusive. Therefore, we performed this systematic review and meta-analysis to provide a robust portfolio of evidence to identify and update the association of probiotics with cirrhosis and to explore the potential mechanistic actions and biological effects. We also discuss the results of the interaction of the intestinal microbiota, its related metabolites, and inflammatory factors with liver cirrhosis.

## 2 Methods

### 2.1 Protocol registration

The protocol for this systematic review and meta-analysis was registered in advance with PROSPERO (CRD42023386931). The process of systematic evaluation and meta-analysis was recorded in the PRISMA 2020 checklist (Table S1).

### 2.2 Search strategy

A total of 561 potentially eligible references were identified through electronic and manual searches. Relevant articles published from 2008 to 31 October 2022 with no restriction on English language were searched in the following electronic databases: CNKI, Wanfang Data, Web of Science, PubMed, Embase, Medline, and Cochrane Library. We used the following search strategy: ((((((Liver Cirrhosis[MeSH Terms]) OR (Hepatic Cirrhosis[Title/Abstract])) OR (Cirrhosis, Hepatic[Title/Abstract])) OR (Cirrhosis, Liver[Title/Abstract])) OR (Fibrosis, Liver[Title/Abstract])) OR (Liver Fibrosis[Title/Abstract])) AND ((((((Probiotics[MeSH Terms]) OR (Probiotic[Title/Abstract])) OR (Synbiotics[MeSH Terms])) OR (Synbiotic[Title/Abstract])) OR (Prebiotics[MeSH Terms])) OR (Prebiotic[Title/Abstract])). Moreover, bibliographies of all relevant prior reviews identified by the search strategy were scanned for a comprehensive literature search. Two researchers (Lei and Xia) screened the search outcomes to identify relevant studies for this systematic review and meta-analysis.

### 2.3 Inclusion and exclusion criteria

The inclusion criteria for selecting eligible studies were as follows: (1) studies provided a measure of inflammatory factors and/or liver function index, (2) studies involved patients with a definite diagnosis of cirrhosis, and (3) study had to be a randomized controlled trial (RCT) comparing the use of probiotics with a placebo or no intervention.

The exclusion criteria were as follows: (1) studies were literature reviews, animal experiments, no control trials, non-RCTs, low-quality literature, and case reports; and (2) patients had the antibiotic before their measurements.

### 2.4 Data extraction

Data extraction from eligible literature was performed by two investigators (Lei and Xia) following the guidelines laid out in the QUOROM statement [71]. All data were integrated into a standard form and cross-checked independently by two reviewers; in the event of any disagreement, the disputes shall be submitted to a third reviewer for assistance in arbitration. Data related to the following variables were extracted from each targeted study: (1) the last name of first author and year of publication; (2) sample size and duration of study; (3) participants’ baseline information such as gender and age; (4) composition and dose of supplements for the experimental group – the experimental group is based on the control group with the addition of microecological preparations (e.g., *Bifidobacterium* triplex capsules, *Bifidobacterium* quadruplex tablets); and (5) treatment of the control group – the control group is usually treated with conventional treatment of cirrhosis (e.g., hepatic protection, diuretic, anti-infective treatment, timely replenishment of albumin and plasma, and symptomatic treatment of patients).

### 2.5 Statistical analysis

Statistical analysis of data was performed using software RevMan Version 5.4 (The Cochrane Collaboration, Copenhagen, Denmark). For dichotomous variables, the rate difference (RD) and its 95% confidence interval (95% CI) were chosen as effect sizes; for continuous variables, the weighted mean difference was used if the measurement units were consistent, and the standardized mean difference and its 95% CI were used as effect sizes if the measurement units were inconsistent. The chi-squared test was used to assess the presence of statistical heterogeneity, and the *I* ^2^ test was used to evaluate the magnitude of heterogeneity. If there was no statistical heterogeneity among the results of the studies (*P* ≥ 0.05 and *I* ^2^ ≤ 50%), a fixed-effects model was used for meta-analysis; if the heterogeneity was large (*P* < 0.05 and *I* ^2^ > 50%), a random-effects model was used and the possible causes were analyzed, and meta-analysis was considered with subgroup analysis and sensitivity analysis. The methodological quality of the included studies was evaluated using the risk of bias assessment tool for RCTs recommended by the Cochrane Handbook for Systematic Reviews of Interventions 6.3, and publication bias was analyzed using a funnel plot (Figures S1–S9). A *P*-value of <0.05 was considered a statistically significant difference.

### 2.6 Risk of bias (quality) assessment

**Figure 1:**
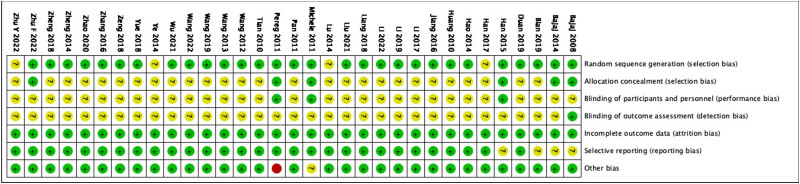
Risk of bias summary: review authors’ judgments about each risk of bias item for each included study.

**Figure 2:**
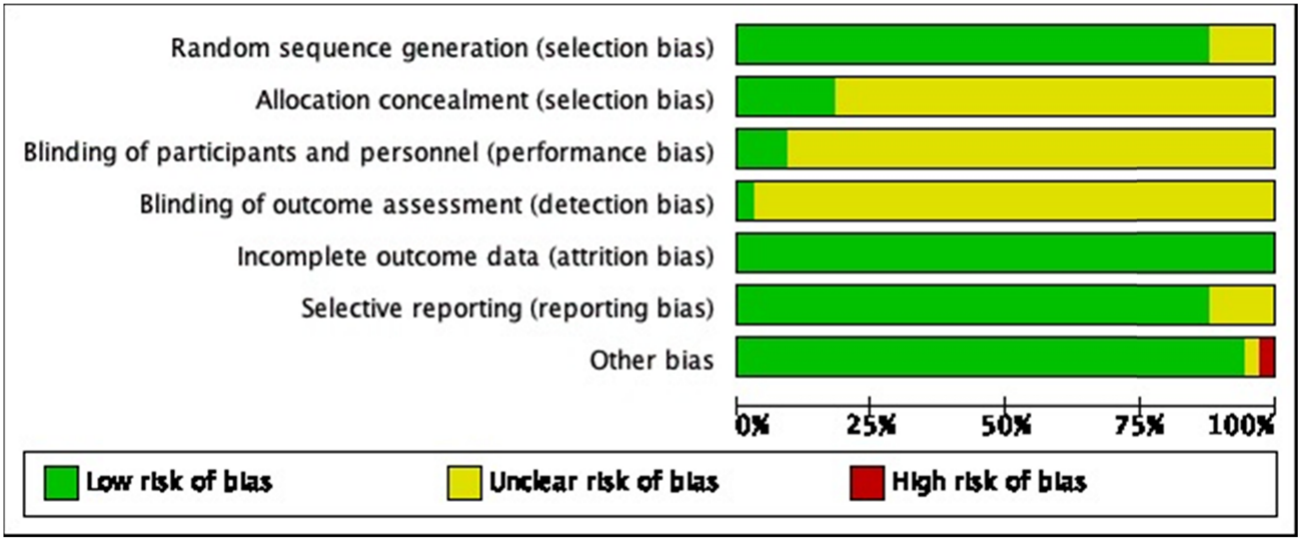
Risk of bias graph: review authors’ judgments about each risk of bias item presented as percentages across all included studies.

Jadad score was used to assess bias of included studies (Figures 1 and 2), based on the following parameters: random sequence generation, allocation concealment, blinding of participants and personnel, incomplete outcome data, selective reporting, and other bias. Two researchers independently evaluated each article that fulfilled the eligibility criteria and resolved disagreements by consensus or by referring to a third investigator.

## 3 Results

### 3.1 Study selection

**Figure 3:**
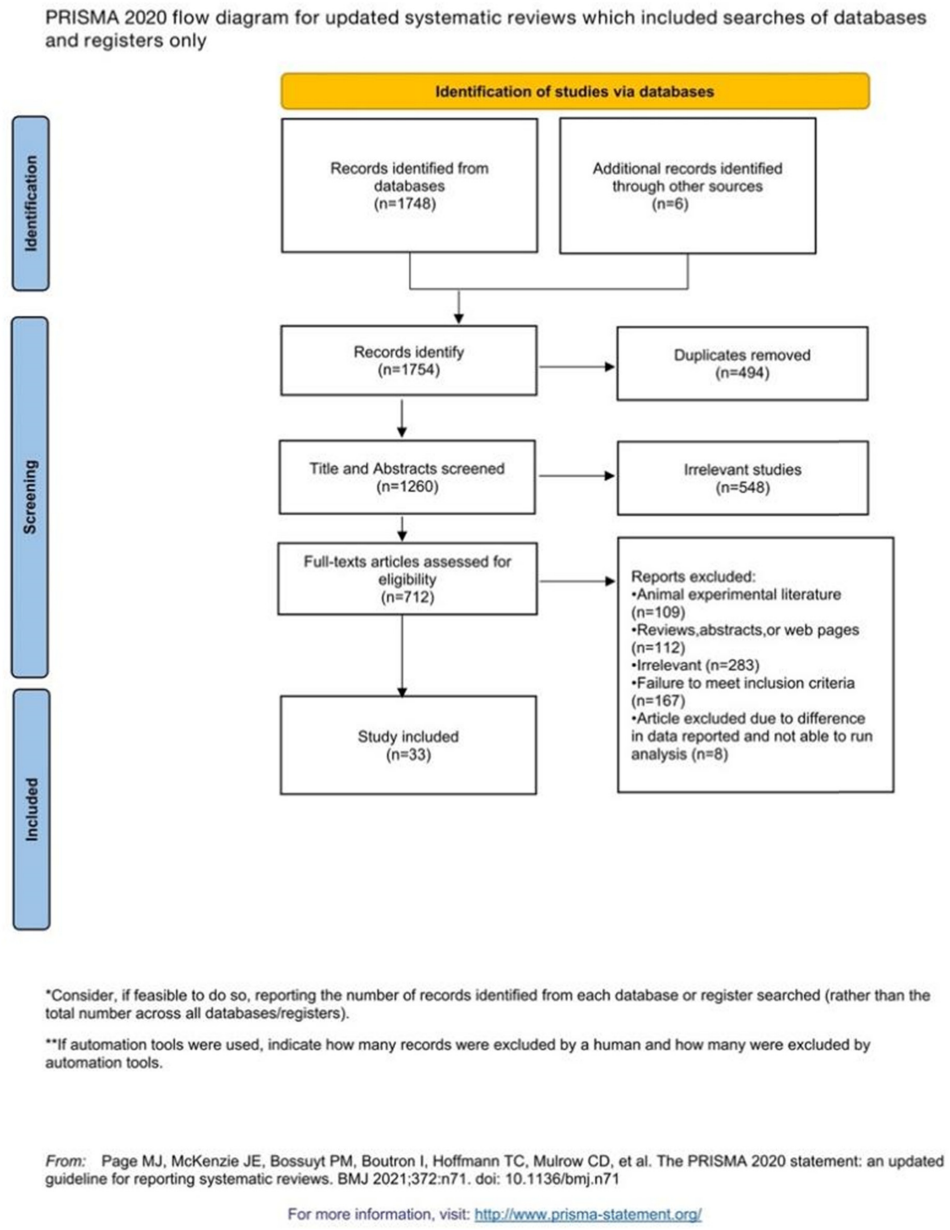
Flow diagram of the study selection procedure.

Figure 3 shows a flow chart for study selection. Among the 1,754 articles identified by the search strategy in the databases, 494 were excluded because of duplication. Title and abstract screening resulted in 548 irrelevant articles removed. The full texts of the remaining 712 articles were reviewed with respect to the study selection criteria, and this process disqualified 679 articles. Eventually, a total of 33 studies were included in this systematic review and meta-analysis.

### 3.2 Study characteristics

A detailed description of the characteristics (author, year, sample size, age, sex, intervention, duration of trial, main outcomes, and Jaded score) of the 33 eligible studies is summarized in Table 1. We selected 33 RCTs for our final analysis. The control group included 1,367 participants, whereas the experimental group included 1,045 participants. In the literature with relevant data, there were no statistically significant differences between the two groups in terms of age and gender. The trial durations ranged from 1 to 48 weeks. The included studies were published between 2008 and 2022, with sample sizes ranging from 20 to 298.

**Table 1:** Main characteristics of studies included in the systematic review and meta-analysis (*n* = 33)

Study	Participants		Age (mean ± SD)	Male/female	Intervention		Duration of trail (in weeks)	Outcome	Jadad score
	Experimental	Control			Experimental	Control			
Bajaj et al. (2008) [142]	14	6	E: 52 ± 8		Yogurt with *L. bulgaricus* and *S. thermophilus*	Placebo	8	Ammonia, IL-6, TNF-α	5
			C: 54 ± 4						
Pereg et al. (2011) [143]	18	18	E: 63.2 ± 10.5		*Lactobacillus acidophilus, L. bulgaricus, B. bifidum,* and *Streptococcus thermophiles*	Placebo (non-fermentable fibers)	24	Albumin, AST, ALT, ammonia, INR	6
			C: 65.9 ± 8.4						
Bajaj et al. (2014) [118]	14	16	E: 58.4 ± 3.8	E: 10/4	*Lactobacillus GG*	Placebo	8	INR, bilirubin, ammonia, albumin, endotoxin, TNF-α, IL-6, IL-2, IL-1β, IL-10, IL-17	5
			C: 58.5 ± 4.5	C: 12/4					
Han et al. (2015) [144]	60	57	52.7 ± 11.3	75/42	*Lactobacillus subtilis* and *Streptococcus faecium* + conventional therapy	Conventional therapy	1	Total protein, albumin, AST, ALT, ALP, yGT, TB, cholesterol, PT	6
Malaguarnera et al. (2011) [145]	34	32	E: 46.9 ± 5.4	E: 18/16	*Bifidobacterium longum* + conventional therapy and ofloxacin	Conventional therapy and ofloxacin	24	ALT, AST, bilirubin, albumin, TNF-α	6
			C: 46.7 ± 5.7	C: 15/17					
Feng et al. (2010) [146]	13	9	E: 57 ± 12	E: 10/3	*B. bifidum, L. acidophilus,* and *E. faecalis* + conventional therapy	Conventional therapy	12	Endotoxin, IL-2, IL-6, TNF-α	4
			C: 53 ± 11	C: 7/2					
Huang et al. (2010) [147]	15	15			*Clostridium butyricum* + conventional therapy and ofloxacin	Conventional therapy and ofloxacin	2	Endotoxin, TNF-α, IL-6, PAF	4
Pan and Huang (2011) [148]	34	32	E: 53 ± 7	E: 20/14	*B. bifidum, L. acidophilus,* and *E. faecalis* + conventional therapy	Conventional therapy	4	Endotoxin, ALT, AST, IL-2, IL-6, TNF-α	4
			C: 52 ± 7	C: 18/14					
Wang et al. (2012) [149]	30	30			*B. bifidum, L. acidophilus, E. faecalis,* and *Bacillus cereus* + conventional therapy	Conventional therapy	4	IL-1α, IL-6, TNF-α	4
Wang and Li (2013) [150]	32	24	E: 47 ± 8	E: 19/13	*Bacillus subtilis* and *E. faecium* + antiviral treatment	Antiviral treatment	4	Endotoxin, TNF-α	4
			C: 48 ± 4	C: 16/8					
Lu (2014) [151]	17	18			*B. subtilis* and *E. faecium* + conventional therapy	Conventional therapy	4	Endotoxin, TNF-α	3
Ye and Xiang (2014) [152]	42	42	E: 68.5 ± 7.1	E: 23/19	*B. bifidum, L. acidophilus,* and *E. faecalis* + conventional therapy	Conventional therapy	4	hs-CRP, TNF-α, efficacy	3
			C: 69.0 ± 7.3	C: 25/17					
Zheng et al. (2014) [153]	45	40	45.2 ± 13.5	62/23	*B. bifidum, L. acidophilus, E. faecalis,* and *cephalosporin* + conventional therapy cephalosporin combined with quinolone	Conventional therapy cephalosporin combined with quinolone	2	ALT, AST, albumin, PTA, endotoxin, TNF-α, IL-2, IL-6, IL-10, efficacy	4
Hao et al. (2014) [154]	32	31	E: 41.6 ± 6.8	E: 18/14	*B. bifidum, L. acidophilus,* and *E. faecalis* + conventional therapy	Conventional therapy	12	Endotoxin, TNF-α, IL-6	4
			C: 42.3 ± 5.4	C: 16/16					
Zhang (2016) [155]	52	52	E: 40.7 ± 8.5	E: 33/19	*B. bifidum, L. acidophilus,* and *E. faecalis* + antiviral treatment	Antiviral treatment	24	HBV DNA, ALT, AST, albumin, TBIL, efficacy	4
			C: 40.8 ± 9.1	C: 34/18					
Jiang and Xie (2016) [156]	32	32	E: 54.4 ± 7.12	E: 21/11	*B. bifidum, L. acidophilus,* and *E. faecalis* + Conventional therapy	Conventional therapy	2	DAO, ammonia, endotoxin, ALT, AST, TBIL, albumin, PT	4
			C: 58.2 ± 7.12	C: 24/8					
Li et al. (2017) [157]	40	40	E: 35.8 ± 4.8	E: 18/22	*L. acidophilus, E. faecalis, B. subtilis* + *Fluoroquinolone*	Fluoroquinolone	48	TNF-α, IL-6, ALT, AST, AKP, GGT, albumin, efficacy	4
			C: 36.5 ± 3.5	C: 17/23					
Han and Bai (2017) [158]	49	49			*B. bifidum, L. acidophilus,* and *E. faecalis* + conventional therapy	Conventional therapy	2	TNF-α, IL-6, IL-10, endotoxin, AST ALT albumin, efficacy	3
Zheng et al. (2018) [159]	100	100	E: 47.28 ± 7.24	E: 63/37	*B. bifidum, L. acidophilus,* and *E. faecalis* + Conventional therapy	Conventional therapy	2	AST, ALT, albumin, TNF-α, IL-2, IL-6, IL-10, efficacy	4
			C: 46.38 ± 7.04	C: 60/40					
Yue and Fang (2018) [160]	40	40	E: 52.11 ± 9.43	E: 23/17	*B. bifidum, L. acidophilus, E. faecalis,* and *B. cereus* + Conventional therapy	Conventional therapy	8	ALT, AST, albumin, PCT, endotoxin	4
			C: 53.12 ± 10.45	C: 22/18					
Zeng et al. (2018) [161]	80	80	E: 45.06 ± 6.72	E: 41/39	*B. bifidum, L. acidophilus,* and *E. faecalis* + Conventional therapy	Conventional therapy	24	ALT, AST, TBIL, ammonia, endotoxin	4
			C: 43.62 ± 6.29	C: 45/35					
Liang and Chen (2018) [162]	28	28	E: 44.2 ± 6.2	E: 17/11	*B. bifidum, L. acidophilus, E. faecalis,* and *B. cereus* + Conventional therapy	Conventional therapy	24	AST, ALT, TBIL	4
			C: 44.3 ± 6.5	C: 15/13					
Wang and Wu (2019) [163]	149	149	E: 50.93 ± 4.26	E: 103/46	*B. bifidum, L. acidophilus, E. faecalis* + Conventional therapy and entecavir	Conventional therapy and entecavir	12	TBIL, ALT, AST, LPS, TNF-α	4
			C: 51.62 ± 3.97	C: 98/51					
Li et al. (2019) [164]	57	57	E: 69.54 ± 3.31	E: 37/20	*B. bifidum, L. acidophilus, E. faecalis* + *lamivudine* and *adefovir dipivoxil*	Lamivudine and adefovir dipivoxil	24	ALT, AST, TBIL	4
			C: 69.79 ± 3.47	C: 35/22					
Duan (2019) [165]	42	42	E: 45.24 ± 7.38	E: 26/16	*B. bifidum, L. acidophilus,* and *E. faecalis +* Antiviral treatment	Antiviral treatment	24	ALT, AST, TBIL	4
			C: 46.17 ± 6.59	C: 24/18					
Bian et al. (2019) [166]	45	45	E: 57.12 ± 4.02	E: 26/19	*B. bifidum, L. acidophilus,* and *E. faecalis* + Conventional therapy	Conventional therapy	2	ALT, AST, TBIL, IL-10, TNF-α, endotoxin	4
			C: 56.98 ± 4.01	C: 24/21					
Zhao and Zhao (2020) [167]	49	49	E: 48.07 ± 6.98	E: 33/16	*B. bifidum, L. acidophilus, E. faecalis,* and *B. cereus* + Conventional therapy	Conventional therapy	1	IL-6, IL-8, TNF-α	4
			C: 47.33 ± 6.85	C: 30/19					
Liu and Li (2021) [168]	30	30	E: 53.5 ± 11.7	E: 18/12	*Bacillus licheniformis* + Conventional therapy	Conventional therapy	4	IL-2, IL-6, TNF-α, TBIL, ALT, AST	4
			C: 52.3 ± 12.1	C: 16/14					
Wu et al. (2021) [169]	50	50	E: 44.32 ± 6.5	E: 31/19	*B. bifidum, L. acidophilus,* and *E. faecalis* + Conventional therapy	Conventional therapy	2	ALT, AST, TBIL, IL-10, TNF-α, endotoxin	4
			C: 43.63 ± 6.82	C: 32/18					
Zhu et al. (2022) [170]	35	35	E: 55.63 ± 2.38	E: 21/14	*B. bifidum, L. acidophilus, E. faecalis,* and *B. cereus* + Conventional therapy	Conventional therapy	2	Efficacy, AST, ALT, TBIL	5
			C: 55.47 ± 2.16	C: 20/15					
Li et al. (2022) [171]	50	54	51.82 ± 12.46		*Yogurt with B. bifidum, L. rhamnosus, L. acidophilus, L. bulgaricus,* and *S. thermophilus* + Conventional therapy	Conventional therapy	3	IL-1β, IL-6, TNF-α, ALT, AST, GGT, ALP	4
Zhu et al. (2022) [172]	42	30	E: 46.12 ± 3.6	E: 20/22	*B. subtilis, E. faecium* + Conventional therapy	Conventional therapy	4	Efficacy, AST, ALT, TBIL, albumin	3
			C: 46.41 ± 3.55	C: 14/16					
Wang and Jin (2022) [173]	35	35	52.38 ± 8.19	39/31	*B. bifidum, L. acidophilus, E. faecalis,* and *B. cereus* + Conventional therapy	Conventional therapy	2	Efficacy, TBIL, ALT, AST, endotoxin	4

### 3.3 Main outcomes

#### 3.3.1 Efficacy

**Figure 4:**
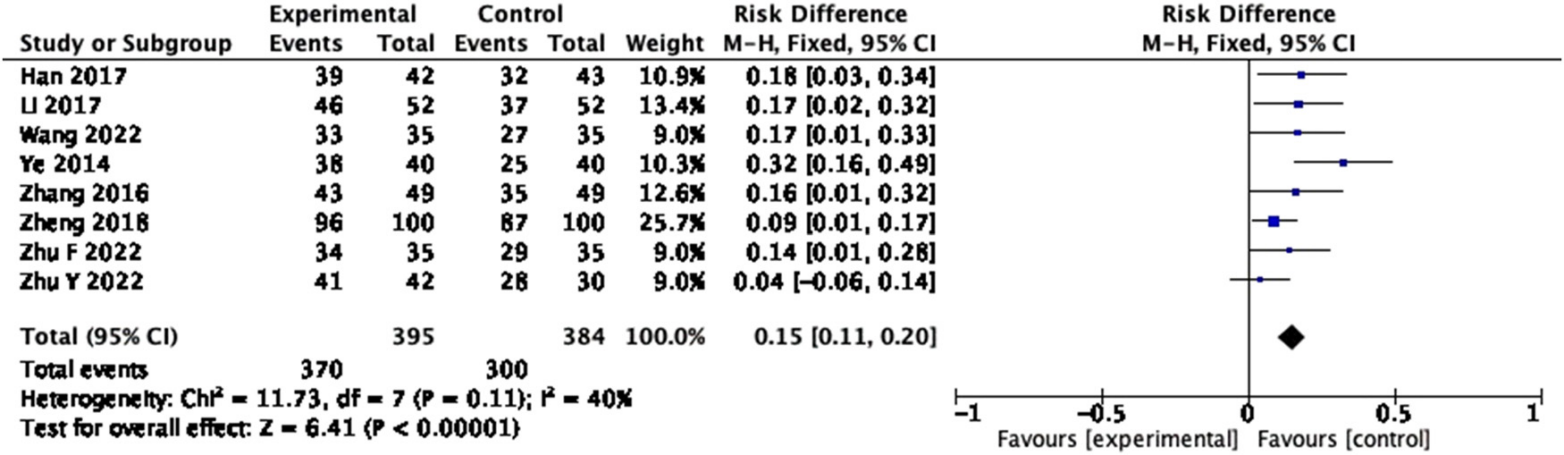
Forest plot of efficacy of probiotics intervention in cirrhosis (fixed-effects model).

A total of eight studies reported efficacy, defining significant and effective as “effective.” The homogeneity among the studies was good (*P* = 0.11, *I* ^2^ = 40%); thus, the fixed-effects model was used. The forest plot showed that the total clinical effective rate of the treatment group was superior to that of the control group, indicating that the probiotic intervention could improve the total clinical efficiency of patients with cirrhosis to some extent (Figure 4: RD = 0.15, 95% CI = 0.11–0.20, *P* < 0.00001).

#### 3.3.2 Aspartate transaminase (AST)

**Figure 5:**
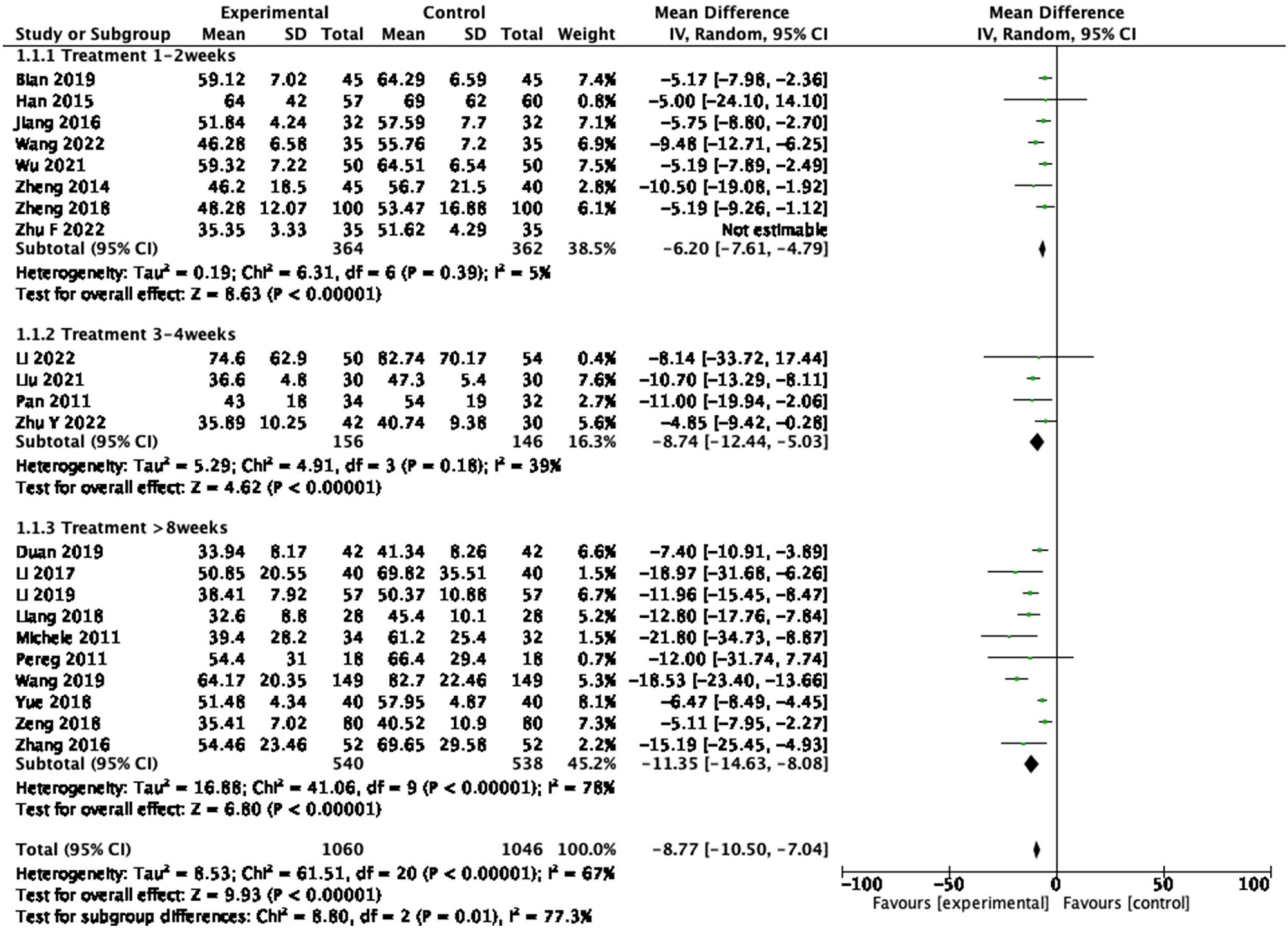
Forest plot of AST between experimental group and control group and subgroup analysis by duration of trail (random-effects model).

A total of 22 studies reported the change of AST level, including 2,106 participants. A pooled analysis using the random-effects model showed large statistical differences between the studies (*P* < 0.001, *I* ^2^ = 84%), so the sensitivity analysis was performed, and heterogeneity decreased after exclusion of Zhu (2022). The forest plot proved that the level of AST was significantly lower in the treatment group (Figure 5: mean deviation (MD) = −8.77, 95% CI = −10.50 to −7.04, *P* < 0.00001), indicating that probiotics supplement was effective in reducing AST in patients with cirrhosis. Further subgroup analysis by duration of trail showed that the homogeneity was best at 1–2 weeks of treatment (*P* = 0.39, *I* ^2^ = 5%).

#### 3.3.3 ALT

**Figure 6:**
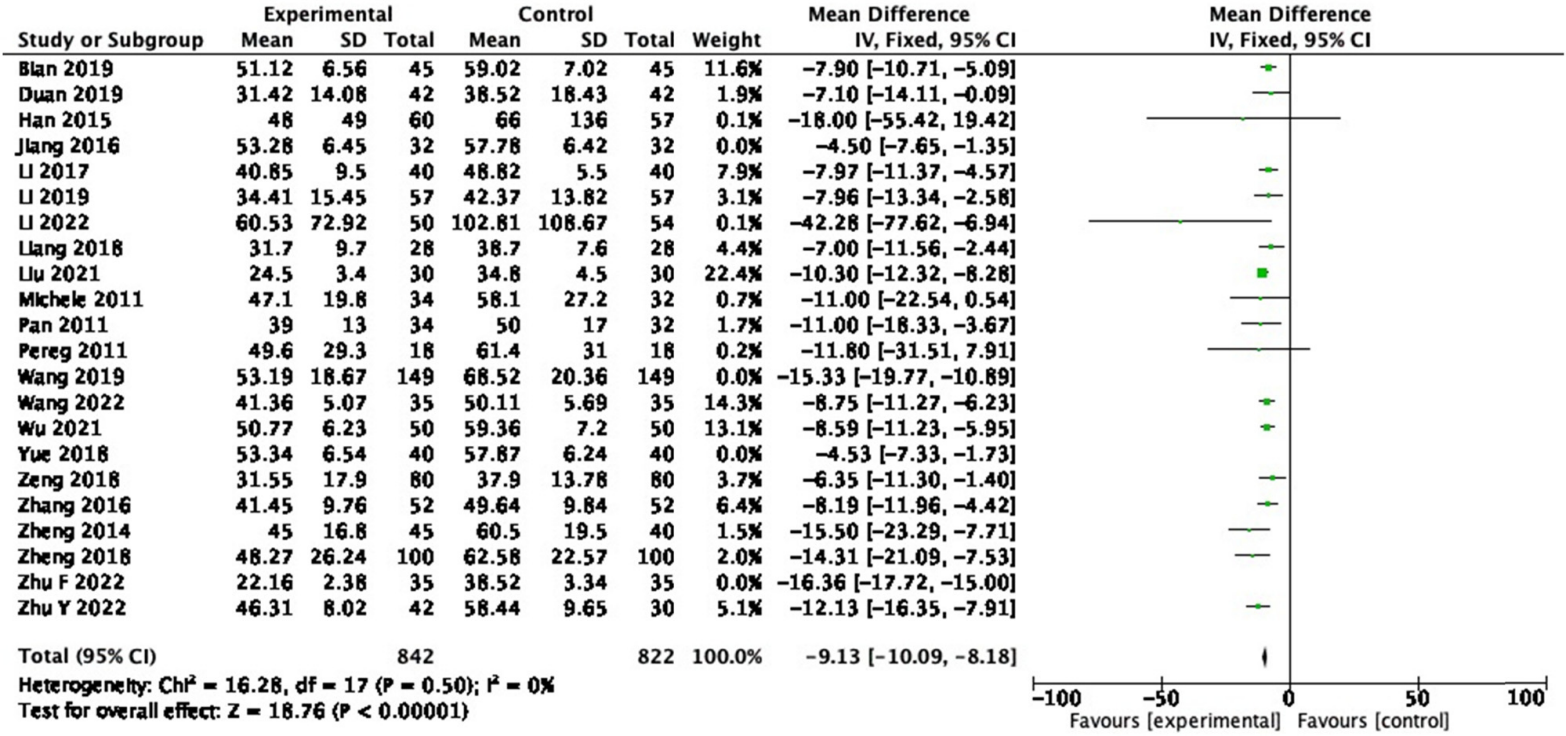
Forest plot of ALT between experimental group and control group (random-effects model).

From 23 RCTs reporting on ALT, 1,664 participants were included. In the pooled analysis of studies, a significant effect of probiotic on ALT reduction (Figure 6: MD = −9.13, 95% CI = −10.09 to −8.18, *P* < 0.00001) was observed with a heterogeneity of 84% (*P* < 0.001). After the sensitivity analysis was performed, four articles, namely, Jiang (2016), Wang (2019), Yue (2018), and Zhu F (2022) were excluded, and the homogeneity among the study groups was good (*P* = 0.50, *I* ^2^ = 0%).

#### 3.3.4 IL-6

**Figure 7:**
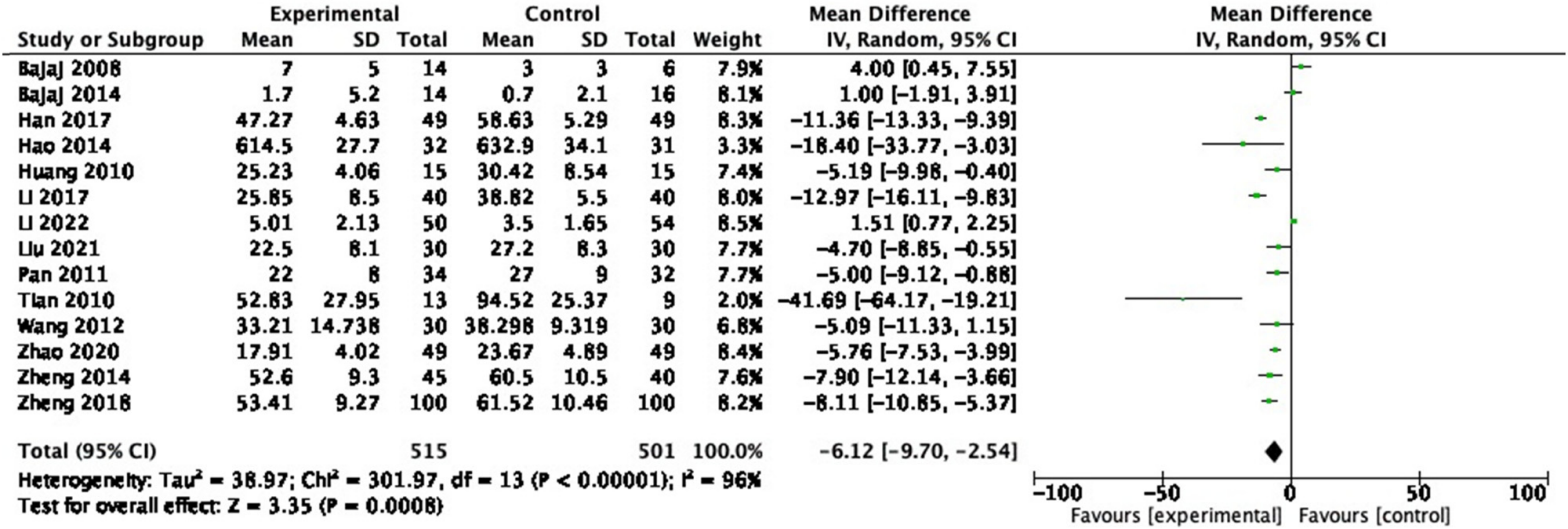
Forest plot of IL-6 between experimental group and control group (random-effects model).

In the 14 RCTs reporting IL-6, 1,016 participants were included. A random-effects meta-analysis of these studies revealed that probiotics decreased IL-6 (Figure 7: MD = −6.12, 95% CI = −9.70 to −2.54, *P* < 0.001) with a heterogeneity of 96% (*P* < 0.00001). Sensitivity analysis and subgroup analysis were performed, and no source of heterogeneity could be found.

#### 3.3.5 TNF-α

**Figure 8:**
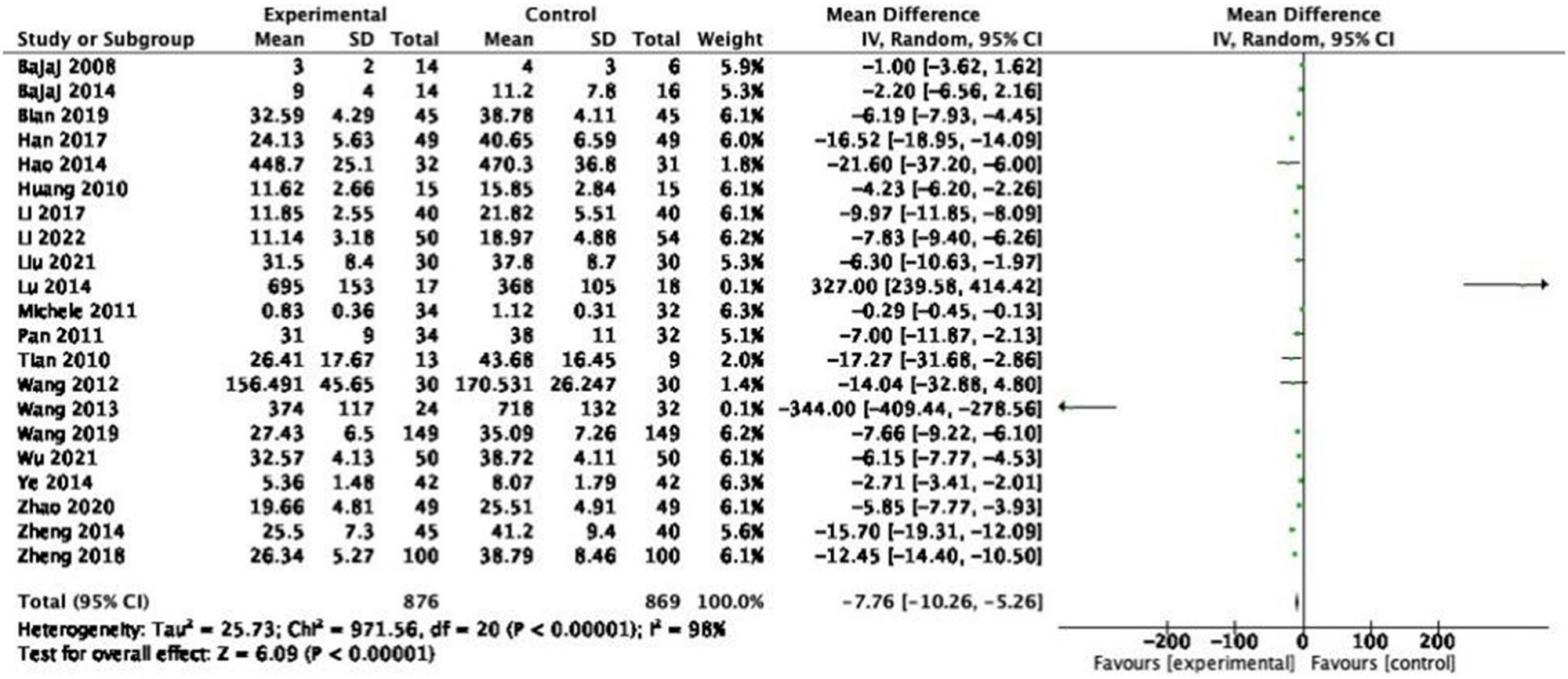
Forest plot of TNF-α between experimental group and control group (random-effects model).

A total of 21 studies reporting the mean change in TNF-α, including 1,745 participants. A random-effects model was used for pooled analysis, and the results demonstrated that the TNF-α levels of patients in the experimental group were lower than those in the control group (Figure 8: MD = −7.76, 95% CI = −10.26 to −5.26, *P* < 0.00001), with a heterogeneity of 98% (*P* < 0.00001). Sensitivity analysis and subgroup analysis were performed, and no source of heterogeneity could be found.

#### 3.3.6 Total bilirubin (TBIL)

**Figure 9:**
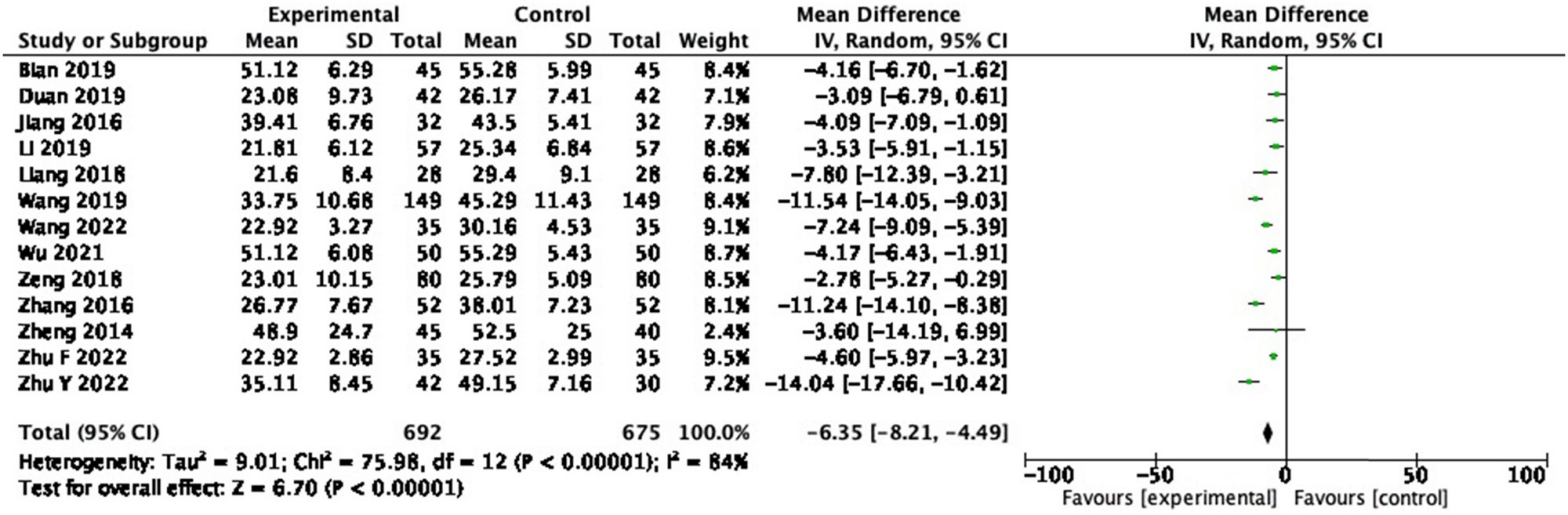
Forest plot of TBIL between experimental group and control group (random-effects model).

In the 13 RCTs reporting TBIL, 1,367 participants were included. A random-effects meta-analysis of these studies illustrated that probiotics decreased TBIL (Figure 9: MD = −6.35, 95% CI = −8.21 to −4.49, *P* < 0.00001) with a heterogeneity of 84% (*P* < 0.00001). Sensitivity analysis and subgroup analysis were performed, and no source of heterogeneity could be found.

#### 3.3.7 Endotoxin

**Figure 10:**
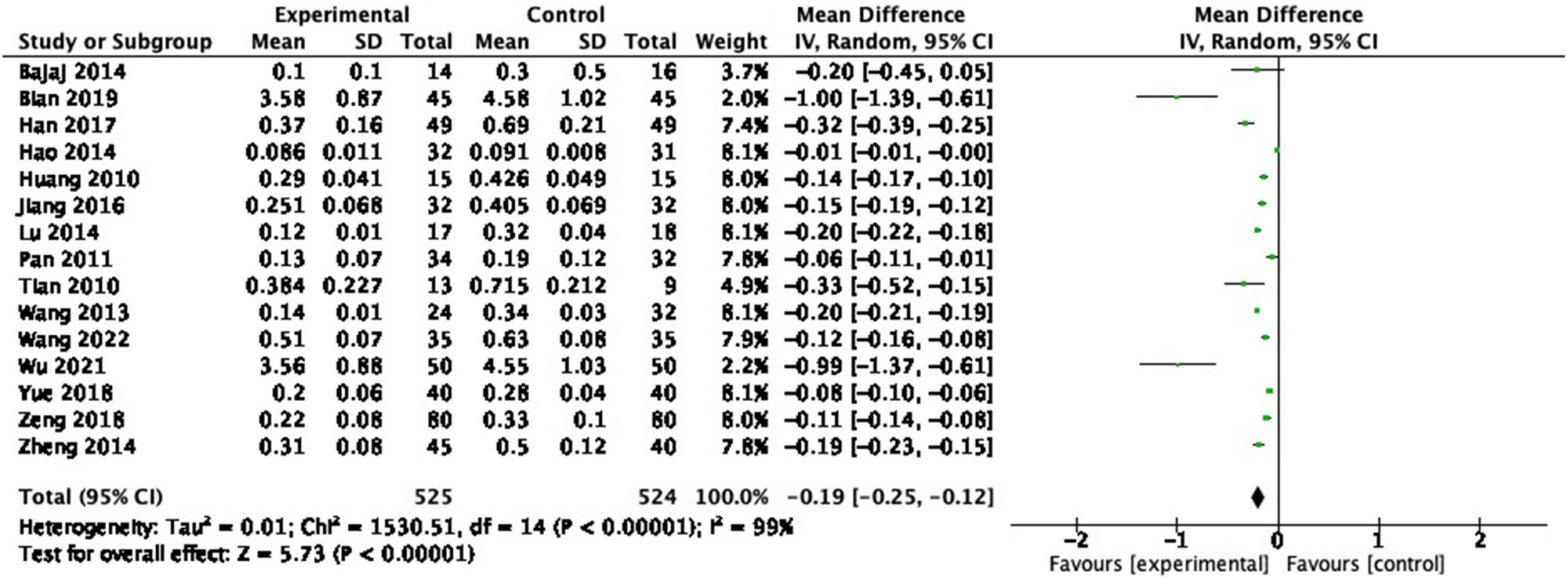
Forest plot of endotoxin between experimental group and control group (random-effects model).

Endotoxin was evaluated in 15 RCTs, and 766 participants were included. In the pooled analysis of studies, a significant effect of probiotic on endotoxin reduction (Figure 10: MD = −0.19, 95% CI = −0.25 to −0.12, *P* < 0.00001) was observed with a heterogeneity of 99% (*P* < 0.00001). Sensitivity analysis and subgroup analysis were performed, and no source of heterogeneity could be found.

#### 3.3.8 IL-2

**Figure 11:**
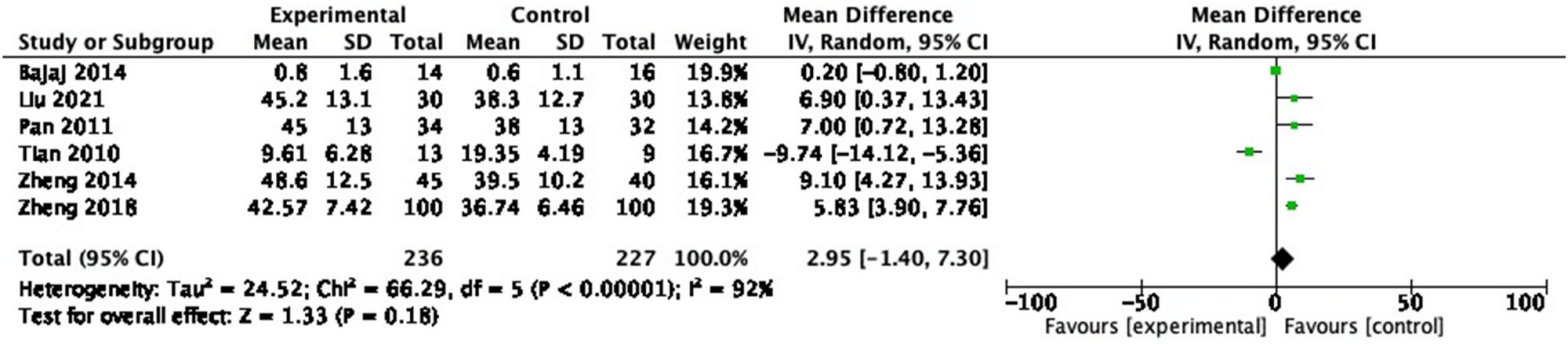
Forest plot of IL-2 between experimental group and control group (random-effects model).

In the six RCTs reporting IL-2, 463 participants were included. In the pooled analysis, no significant change in IL-2 (Figure 11: MD = 2.95, 95% CI = −1.40 to 7.30, *P* = 0.18) was observed with a heterogeneity of 92% (*P* < 0.00001).

#### 3.3.9 IL-10

**Figure 12:**
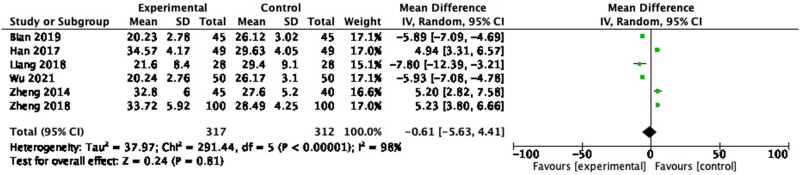
Forest plot of IL-10 between experimental group and control group (random-effects model).

Six RCTs measured IL-10, including 629 participants. In the pooled analysis, no significant effect of probiotic on IL-10 (Figure 12: MD = −0.61, 95% CI = −5.63 to 4.41, *P* = 0.81) was observed with a heterogeneity of 98% (*P* < 0.00001).

## 4 Discussion

Cirrhosis is the common end-stage pathological pathway of liver damage arising from a multitude of chronic liver diseases [72], but its potential mechanisms are far from being elucidated. Cirrhosis develops after a prolonged period of inflammation, leading to the replacement of healthy liver parenchyma with regenerative nodules and fibrotic tissue, resulting in portal hypertension. Numerous types of cells, cytokines, and miRNAs might be involved in the initiation and development of cirrhosis.

This study suggests that cirrhosis may interact with microbiota, related metabolites, and inflammatory factors. Furthermore, this study evaluated the efficacy of probiotic therapy in improving liver function and immunity in patients with cirrhosis. As the largest, most complex and most important microecosystem in the human body, the intestinal microecosystem is known as the second human genome. More than 90% of healthy adults have an intestinal microbiota composed of four major components: *Bacteroidetes* , *Firmicutes* , *Proteobacteria* , and *Actinobacteria* [73]. By contrast, the diversity and proportion of intestinal microorganisms in patients with cirrhosis are different from those in healthy individuals.

A growing amount of recent evidence has elaborated on the altered composition of the gut microbiota in patients with cirrhosis, which is mainly characterized by a shortage of autochthonous non-pathogenic bacteria and an enrichment of potentially pathogenic bacteria [74]. We compiled the relevant literature and found that the intestinal microbiota of patients with cirrhosis contained more *Enterobacteriaceae* , *Streptococcaceae* , *Fusobacteria* , *Proteobacteria* , *Streptococcus,* and *Veillonella* and less *Bifidobacteria* , *Lachnospiraceae* , *Bacteroidetes,* and *Firmicutes* compared to controls [75,76,77,78,79]. Notably, in order to further explore microbial genes associated with cirrhosis, Qin et al. conducted a large-scale RCT on the macrogenomic characterization of intestinal bioactive metabolites and intestinal microbiota in patients with cirrhosis, bringing new insights from next-generation sequencing [79]. The intestinal microbiota contributes to HE, one of the most devastating and clinically challenging sequelae of cirrhosis, as determined by the analysis of enriched modular microbial function in 98 patients and 83 healthy control individuals.

**Figure 13:**
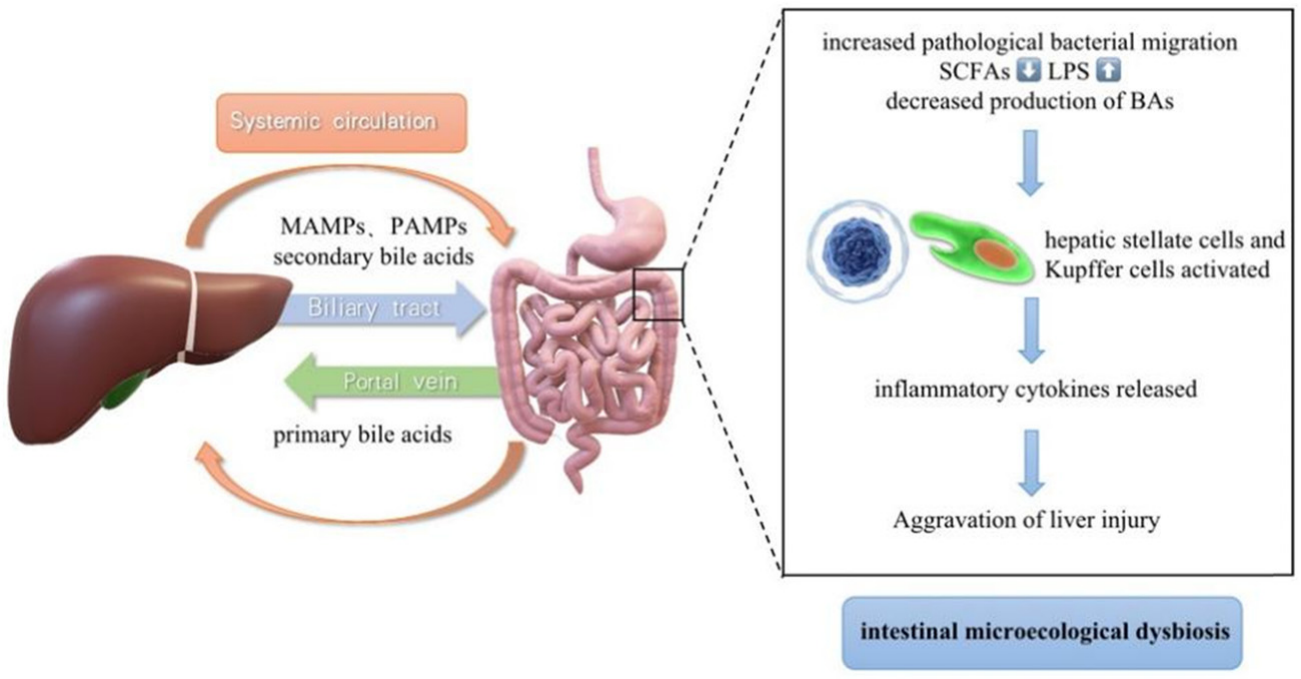
Schematic diagram of the gut–liver axis. Abbreviations: microbial-associated molecular patterns (MAMPs), pathogen-associated molecular patterns (PAMPs), short-chain fatty acids (SCFAs), lipopolysaccharides (LPS), and bile acids (BAs). Three projects that play a prominent role in the relationship between intestinal microbiota and various forms of liver injury are summarized in this study: endotoxin, SCFAs, and BAs [83].

The concept of “gut–liver axis” was brought up to highlight the close functional and anatomical relationship between two organs. The liver is located near the intestine and is supplied with 75% of its blood by the portal vein. The portal venous flow not only supplies the liver with nutrients but also carries an extremely metabolic microbiota of gastrointestinal origin that provides the liver with broad-spectrum antigens. Most of these are commensal products and harmless dietary factors, but translocated pathogens or bacterial-derived factors also enter the liver continuously via the enterohepatic circulation. Under normal circumstances, a handful of bacteria or bacterial metabolites enter the liver, most of which are eliminated by macrophages in the liver (Kupffer cells), with little or no activation. However, in intestinal microecological dysregulation, the intestinal mucosal barrier is damaged due to inflammation or portal hypertension, and increased intestinal permeability leads to increased pathological bacterial migration [80,81]. A multitude of bacteria could thus enter the liver and activate hepatic stellate cells (HSCs) and Kupffer cells. Once these cells are activated, large amounts of pro-inflammatory cytokines are released and involved in liver injury. Increased intestinal permeability of bacteria and their metabolites, known as “leaky gut,” is pervasive in cirrhosis and is a pivotal pathogenic factor leading to major complications [82]. Overall, the liver may be immensely affected by the composition and abundance of intestinal microorganisms, primarily through the receipt of metabolites from microbes (Figure 13).

Bacterial translocation (BT) is defined as the passage of viable bacteria from the intestinal lumen through the mesenteric lymph nodes and other sites [83]. There are at least three mechanisms that have an impact on the pathogenesis of BT: increased intestinal permeability, intestinal bacterial overgrowth, and immune alterations in cirrhosis [84]. The concept of BT was later extended to microbial products, including bacterial DNA, peptidoglycans, and endotoxins [85]. MAMPs of intestinal origin, particularly PAMPs, may elicit or exacerbate innate immune responses in the liver. MAMPs are the basic structures of microorganisms, both pathogenic and non-pathogenic. PAMP includes microbial molecular structures, such as gram-negative-associated LPS.

Endotoxins and LPS are active metabolites of gram-negative bacterial envelope components and pathogenic bacterial derivatives and significantly increase (mainly through the activation of the Toll-like receptor [TLR]4 signaling pathway) the synthesis of a range of inflammatory factors, particularly ILs and TNF-α. Inflammatory cells such as mononuclear macrophages, neutrophils, and lymphocytes release proteases, other enzymes, and reactive oxygen metabolites, contributing to the pathological progression of liver injury. Some hepatic non-immune cells, including endothelial cells and HSC, also have TLRs on their surface that recognize bacterial-specific structural components and subsequently release substantial cytokines such as IL-1, IL-6, and TNF-α, further promoting fibrosis and liver inflammation.

The microbiota produces many metabolites, such as SCFAs and BAs, and these key gut microbial-derived metabolites also continue to shape host immunity and metabolism and are critical in liver injury.

SCFAs serve as an additional source of energy, accounting for 3–9% of our daily caloric intake [86]. SCFAs also have a multitude of functions, from immunomodulation and metabolism to mucosal protection, and therefore have direct and indirect effects on our systemic well-being [87]. Wang et al. implemented *in vivo* and *in vitro* experiments and confirmed that inulin ameliorates inflammation through M1 inhibition and promotion of M2 Mψ induced by SCFAs, which may contribute to the control of liver disease [88]. Profound changes have taken place in the intestinal microbiota of patients with cirrhosis of liver, along with changes in the content of SCFAs. A controlled study demonstrated the functional impact of disturbances in gut microbiota diversity on the production of SCFAs, with significantly lower SCFAs concentrations in cirrhotic patients than in healthy controls, including propionate, butyric acid, and acetic acid, according to functional and differential classification analyses [89].

One of the possible mechanisms of intestinal ecological dysregulation in patients with cirrhosis is related to the reduced secretion of BAs. BAs are signaling molecules that activate specific nuclear farnesoid X receptors (FXRs) in the intestine and activate various signaling pathways by binding to FXR in intestinal epithelial cells and hepatocytes [90]. Approximately 95% of BAs are reabsorbed in the terminal ileum and transported back to the liver via the enterohepatic circulation [91]. However, a small percentage of BAs reach the colon and are converted by the intestinal microbiota into secondary BAs, which regulate various metabolic processes in the liver, such as glucose, triglyceride, cholesterol metabolism as well as inflammatory responses [92,93].

A recent study conducted an extensive analysis of neutrophil function and serum BA composition in a large number of patients with cirrhosis and found that the therapeutic modulation of serum BAs spectrum to “healthy” compositions can restore impaired neutrophil function in patients with cirrhosis, which in turn can reduce bacterial infection and related morbidity and mortality in cirrhosis [94]. This investigator also proposed that BAs’ spectrum regulation could be achieved by targeting the gut microbiome involved in BA metabolism. In addition, except for being a metabolism regulator and a nutrition supply source, bile acid also has an antibacterial function. BAs exert both direct antimicrobial properties and indirect negative selective pressure on intestinal bacteria through FXR activation in the small intestine, which induces antimicrobial peptide synthesis, and FXR agonists reduce BT through the portal pathway to the liver in cirrhosis [92,95].

The gut–liver axis in hepatitis B virus (HBV) and hepatitis C virus (HCV) infections has received much attention recently, and the close association between alterations in the intestinal microbiota and HBV- and HCV-associated complications, such as fibrosis and cirrhosis, is supported by *in vivo* and *in vitro* studies [96,97,98]. Probiotics have an antiviral activity against HBV and HCV infections, and numerous studies have reported mechanisms by which probiotics reduce liver disease by mediating antagonism against HBV and HCV [99]. Lee et al. demonstrated the antiviral activity of *Bifidobacterium adolescentis* SPM0212 against HBV and that probiotic cellular extracts inhibited the replication of HBV viruses by up-regulating the activation of the MxA protein by STAT1 [100]; El-Adawi et al. showed that treatment with probiotic medium extracts significantly reduced HCV viral load and HepG2 cell death; probiotic supplementation attenuated thioacetamide-induced liver cirrhosis in rats by inhibiting the expression of TLR4, CXCL9, and PREX-2 [101]. Recent studies have also suggested that probiotic-adjuvant therapy reduces the risk of further progression to HCC in cirrhotic patients treated with HBC antiviral drugs [102].

In order to assess the *in vitro* resistance of common market probiotics to clinically used antibiotics, Feng et al. isolated and cultured 20 strains of probiotics from nine probiotic preparations and subsequently determined the minimum inhibitory concentration (MIC) of probiotics against 16 antibiotics using the concentration gradient test strip method (E-test) [103]. The results showed that the bacterial probiotics were sensitive to some oral antibiotics – among which, *Bacillus subtilis*, *Lactobacillus *bulgaricus**, *Bifidobacterium *longum**, *Bifidobacterium *infantis,** and *Streptococcus *thermophilus** were sensitive to more than 12 antibiotics, *Enterococcus faecalis* was sensitive to only 5 antibiotics, and fungal probiotic bacteria, such as *Saccharomyces boulardii* CNCMI-45, were resistant to all 16 antibiotics. In the clinical prevention and treatment of antibiotic-associated diseases, attention should be paid to the rational use of probiotics and antibiotics, which can increase the dose of probiotics or stagger the time of medication, preferably at intervals of more than 2–3 h, and does not affect the efficacy of the drug [104].

On the other hand, probiotics can be used in antibiotic therapy in order to reduce the risk of drug-related adverse effects (such as diarrhea) and preserve the clinic’s gut flora [105]. Specific probiotic therapy not only increases the likelihood of eradication but also lessens the negative effects brought on by antibiotic treatment [106]. Potential benefits of using probiotics to treat a variety of diseases include their relatively low cost and the fact that they do not increase the incidence of antibiotic resistance [107,108,109]. Probiotic supplementation therapy has been scientifically demonstrated to increase the effectiveness of antibiotics and preserve host gut flora, and experts hypothesize that adding probiotics to antibiotic therapy may be helpful on top of what is already being done [110]. Therefore, due to the widespread nature of antibiotic resistance and the variety of public health issues it creates, the focused use of minimal side effect drugs, such as probiotics, is particularly crucial [111].

Concerns about the safety of probiotics currently center on [112,113]: can the strains used cause potential infections? Can they carry and transmit drug resistance? Is there regulatory confusion about probiotic products on the market? Can harmful metabolites be produced? The majority of probiotic species, such as *Lactobacillus*, *Bifidobacterium*, *Lactococcus* , and *Yeasts* , fall within the “Generally Recognized as Safe” (GRAS) category [114]. Identification of the origin, antibiotic resistance, purity, potency (amount of live microorganisms given), and final product composition of the strains are among the safety concerns connected with the manufacture of probiotic goods. In order to identify any contaminants, probiotic goods must also be appropriately tested for their intended purpose and shown to be pathogen-free [115]. Numerous extensive systematic studies that looked at hundreds of probiotic trials came to the conclusion that safety problems and adverse events are infrequently documented [116,117]. Probiotics are generally regarded as safe, but caution is advised when using them in immunologically vulnerable populations. Non-industry-sponsored, independent, high-quality, multicenter controlled trials are also required to evaluate the effectiveness and side effects of probiotics in at-risk populations, ideally in conjunction with regulatory agency assessments.

The preceding discussion of this study elucidates the role of the gut microbiota, associated metabolites, and cytokines in the pathophysiology of hepatocyte injury. The results of our meta-analysis showed that the regulation of gut microbiome using probiotics was able to improve the total clinical efficiency of cirrhosis and reduce most of the inflammatory factor indicators. Overall, the enterohepatic circulation, the gut microbiota, and the immune system must establish complex interactions to maintain homeostasis, a disturbance of which can lead to increased intestinal permeability and result in liver disease. It is hoped that this evidence will provide new insights for future research and development of drugs designed to prevent further hepatocyte damage and delay the progression of cirrhosis to hepatocellular carcinoma.

Despite the extensive literature review and rigorous robustness checks, this study comes with a few limitations. First, the heterogeneity of some of the metrics in this meta-analysis is very high, including IL-6, TNF-α, and TBIL. Despite subgroup and sensitivity analyses, unfortunately, the source of heterogeneity could not be found. In view of the inconsistent timeline of the included research and the fact that the participants were from distinct countries and ethnicities, this heterogeneity may be a result of vast individual differences, and therefore, the results in this study for IL-6, TNF-α, and TBIL should be referenced with caution. Second, due to the paucity of relevant literature reporting IL-2 and IL-10, only six studies were included in the analysis of each of these two indicators, and the meta-analysis showed no statistically significant difference. Insufficient attention has been paid to IL-2 and IL-10 in the field of cirrhosis, which indicate potential avenues for further research.

Probiotics not only improve gastrointestinal disorders, but are also beneficial for cirrhosis, as reported in many studies in the literature and confirmed by this meta-analysis [95,118,119,120]. Studies have been conducted to summarize the changes in the gut microbiome of patients with liver disease and discuss the potential role of probiotics in the management of liver disease [83,121]. Alterations in host immune response, host–microbiota metabolism, and intestinal permeability work together in the pathogenesis of cirrhosis, and microbially targeted therapies should focus on increasing potential beneficial taxa and reducing potential harmful taxa.

FMT may shed some light on cirrhosis treatment. As a research hotspot in biomedical and clinical medicine in recent years, FMT is currently the most effective gut microbiota intervention and also an acknowledged treatment for recurrent and refractory *Clostridium difficile* infection (CDI) [122]. This treatment option has received considerable attention since 2013, and as the clinical response of FMT to substantial diseases has been studied in depth [123], its application has been expanded to include not only gastrointestinal diseases (inflammatory bowel diseases, irritable bowel syndrome, CDI) [124,125,126,127,128], but also extra-gastrointestinal disorders (neurological disorders, diabetes, obesity, HE, metabolic disorders, cancer) [129,130,131,132,133,134]. In two noteworthy RCTs on HE, researchers drew the conclusion that FMT treatment is safe and well tolerated in patients with cirrhosis and that it reduces recurrent hospitalizations and improves ecological dysregulation [135,136].

Although the functions and contributions of probiotics in the pathogenesis and complications of cirrhosis remain to be elucidated, these findings could help develop new therapeutic strategies for cirrhosis by focusing on the gut microbiota. Future research should focus on the development of new bioengineering technology-based therapies for cirrhosis that are more efficient and specific [137]: to illustrate, first, the development of artificial compounds that adsorb or modulate specific intestinal microbiota and their metabolites; second, the use of targeted individual microbiota to replace fecal transplants; and finally, the use of engineered microbiota capable of producing anti-inflammatory or antioxidant molecules as an alternative to antibiotics. Notably, it is important that an individualized approach be developed based on the patient’s baseline bowel pattern, biomarkers of response, and other patient factors, with attention to clinically relevant primary outcomes. Further research in this area should delve into personalized approaches, rigorous experimental design, and microbiome treatment selection [133].

The field of liver disease still needs further work on microbial therapies for cirrhosis, including cross-sectional large-scale cohort studies and longitudinal in-depth mechanistic studies to determine the optimal dosing regimen, ideal donor characteristics, duration of efficacy, and benefits and side effects of probiotics. Demographic characteristics and disease-related factors also need to be controlled and taken into account in the interpretation to ensure patient benefits without negative effects on the body [138].

## 5 Conclusions

Albeit there is no effective radical cure for cirrhosis, the results of this systematic review and meta-analysis show that the emerging networks of probiotic interventions, gut microbiota, and inflammatory factors currently prove to be possibly profitable therapeutic targets. The use of probiotics in cirrhosis not only reduced liver transaminase levels but also appeared to improve inflammation and decrease pro-inflammatory markers. Previously published systematic reviews and meta-analyses of the same kind also point to a beneficial effect of probiotic/prebiotic use on liver injury [139,140,141]. Probiotics are relatively safe and well tolerated, which makes them advantageous over other treatment regimens for long periods of time. In the future, microbial therapy may become a potential method and component in the treatment of cirrhosis. Further *in vitro* validation experiments are needed to better understand the mechanism of probiotics in the treatment of cirrhosis. Meanwhile, large-scale population RCTs with homogeneous preparations and the same treatment duration are required to determine whether our results can be generalized to all individuals.

## Supplementary Material

supplementary material
