# Supplementary material for: Probiotic management and inflammatory factors as a novel treatment in cirrhosis: A systematic review and meta-analysis
Source: Open Life Sci. 2023 Oct 17;18(1):20220741. doi: 10.1515/biol-2022-0741 (PMC10590617; doi:10.1515/biol-2022-0741)
Supplement: supplementary material [file biol-2022-0741-sm.pdf]

Supplementary material

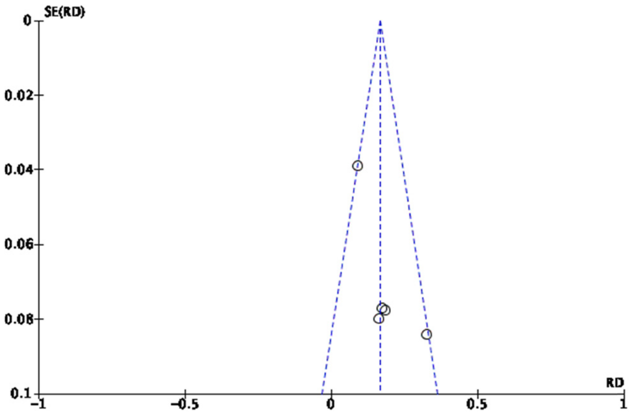

Figure S1: Funnel plot describing publication bias of Efficacy.

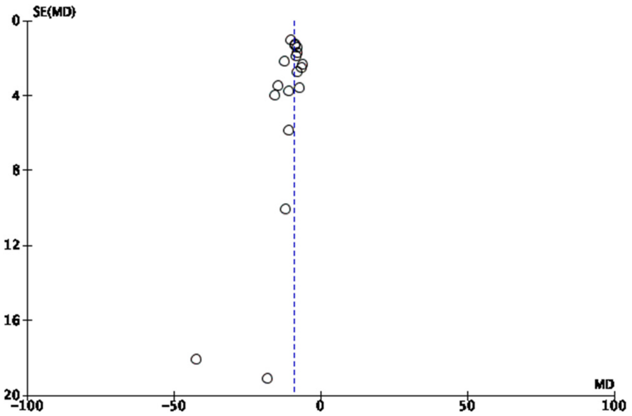

Figure S3: Funnel plot describing publication bias of ALT.

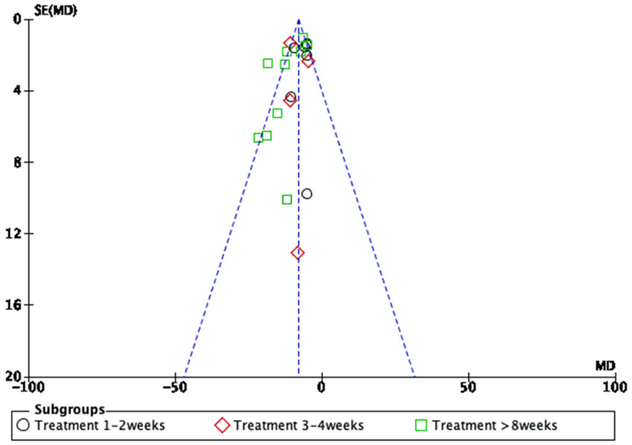

Figure S2: Funnel plot describing publication bias of AST.

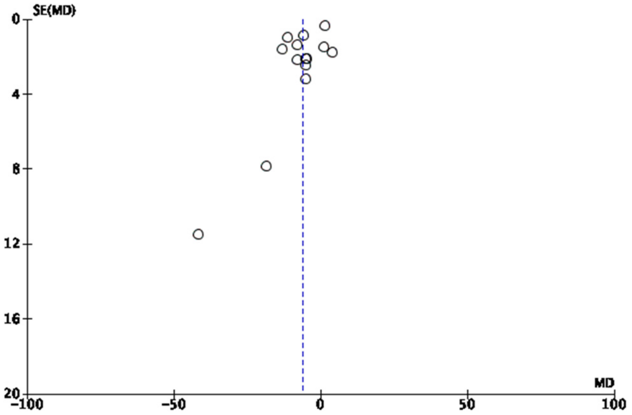

Figure S4: Funnel plot describing publication bias of IL-6.

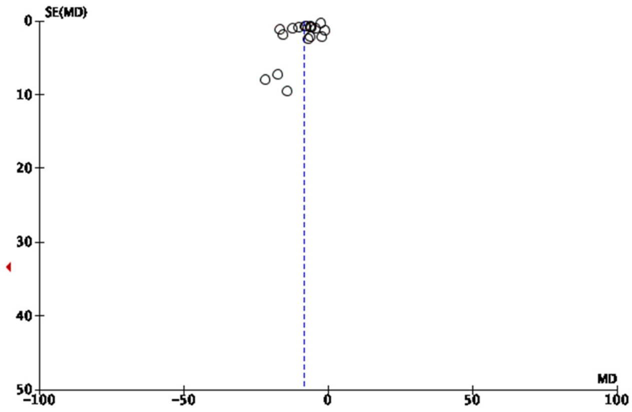

Figure S5: Funnel plot describing publication bias of TNF-α.

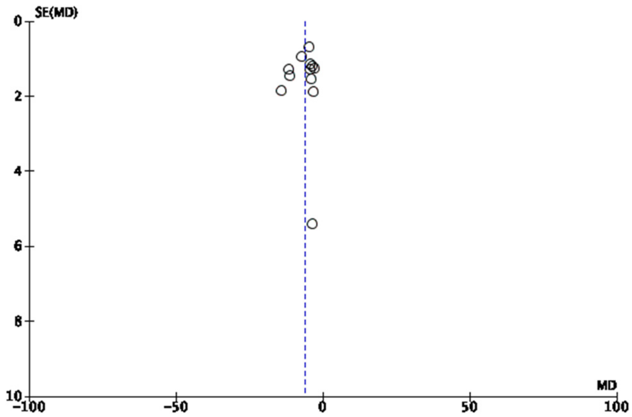

Figure S6: Funnel plot describing publication bias of TBIL.

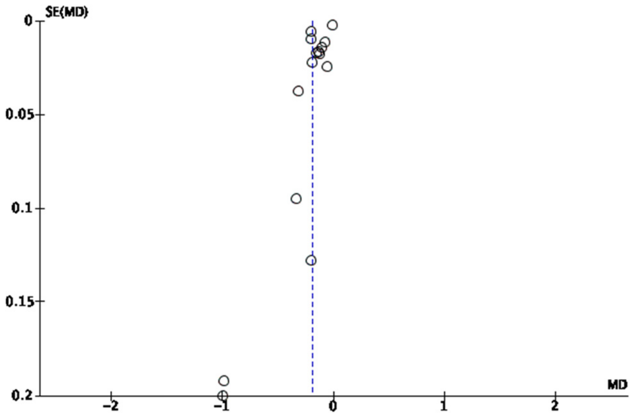

Figure S7: Funnel plot describing publication bias of Endotoxin.

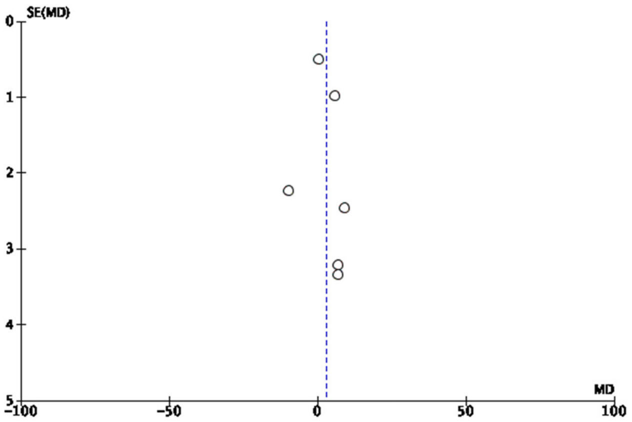

Figure S8: Funnel plot describing publication bias of IL-2.

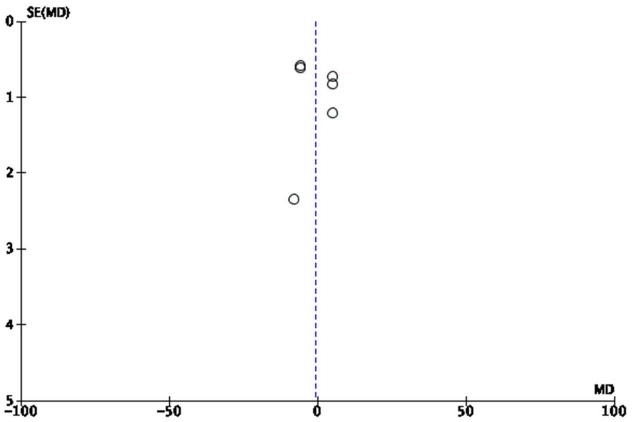

Figure S9: Funnel plot describing publication bias of IL-10.

Table S1: PRISMA Checklist

Section and Topic	Item #	Checklist item	Location where item is reported
**TITLE**			
Title	1	Identify the report as a systematic review	Page 1
**ABSTRACT**			
Abstract	2	See the PRISMA 2020 for Abstracts checklist	Page 1
**INTRODUCTION**			
Rationale	3	Describe the rationale for the review in the context of existing knowledge	Page 2
Objectives	4	Provide an explicit statement of the objective(s) or question(s) the review addresses	Page 2
**METHODS**			
Eligibility criteria	5	Specify the inclusion and exclusion criteria for the review and how studies were grouped for the syntheses	Page5 Section 2.3
Information sources	6	Specify all databases, registers, websites, organisations, reference lists and other sources searched or consulted to identify studies. Specify the date when each source was last searched or consulted	Page 5 Section 2.2
Search strategy	7	Present the full search strategies for all databases, registers and websites, including any filters and limits used	Page 5 Section 2.2
Selection process	8	Specify the methods used to decide whether a study met the inclusion criteria of the review, including how many reviewers screened each record and each report retrieved, whether they worked independently, and if applicable, details of automation tools used in the process	Page 5 Section 2.4
Data collection process	9	Specify the methods used to collect data from reports, including how many reviewers collected data from each report, whether they worked independently, any processes for obtaining or confirming data from study investigators, and if applicable, details of automation tools used in the process	Page 5 Section 2.4
Data items	10a	List and define all outcomes for which data were sought. Specify whether all results that were compatible with each outcome domain in each study were sought (e.g. for all measures, time points, analyses), and if not, the methods used to decide which results to collect	Page 5 Section 2.4
	10b	List and define all other variables for which data were sought (e.g. participant and intervention characteristics, funding sources). Describe any assumptions made about any missing or unclear information	Page 5 Section 2.4
Study risk of bias assessment	11	Specify the methods used to assess risk of bias in the included studies, including details of the tool(s) used, how many reviewers assessed each study and whether they worked independently, and if applicable, details of automation tools used in the process	Page 6 Section 2.6
Effect measures	12	Specify for each outcome the effect measure(s) (e.g. risk ratio, mean difference) used in the synthesis or presentation of results	Page 5 Section 2.5

(Continued)

Table S1: *Continued*

Section and Topic	Item #	Checklist item	Location where item is reported
Synthesis methods	13a	Describe the processes used to decide which studies were eligible for each synthesis (e.g. tabulating the study intervention characteristics and comparing against the planned groups for each synthesis (item #5))	Page 5 Section 2.5
	13b	Describe any methods required to prepare the data for presentation or synthesis, such as handling of missing summary statistics, or data conversions	Page 5 Section 2.5
	13c	Describe any methods used to tabulate or visually display results of individual studies and syntheses	Page 5 Section 2.5
	13d	Describe any methods used to synthesize results and provide a rationale for the choice(s). If meta-analysis was performed, describe the model(s), method(s) to identify the presence and extent of statistical heterogeneity, and software package(s) used	Page 5 Section 2.5
	13e	Describe any methods used to explore possible causes of heterogeneity among study results (e.g. subgroup analysis, meta-regression)	Page 5 Section 2.5
	13f	Describe any sensitivity analyses conducted to assess robustness of the synthesized results	Page 5 Section 2,5
Reporting bias assessment	14	Describe any methods used to assess risk of bias due to missing results in a synthesis (arising from reporting biases)	Page 5 Section 2.6
Certainty assessment	15	Describe any methods used to assess certainty (or confidence) in the body of evidence for an outcome	Page 5 Section 2.5
**RESULTS**			
Study selection	16a	Describe the results of the search and selection process, from the number of records identified in the search to the number of studies included in the review, ideally using a flow diagram	Page 7 Section 3.1
	16b	Cite studies that might appear to meet the inclusion criteria, but which were excluded, and explain why they were excluded	Page 7 Section 3.1
Study characteristics	17	Cite each included study and present its characteristics	Page 7 Section 3.2
Risk of bias in studies	18	Present assessments of risk of bias for each included study	Page 6 Section 2.6
Results of individual studies	19	For all outcomes, present, for each study: (a) summary statistics for each group (where appropriate) and (b) an effect estimate and its precision (e.g. confidence/credible interval), ideally using structured tables or plots	Page 7 Section 3.3
Results of syntheses	20a	For each synthesis, briefly summarise the characteristics and risk of bias among contributing studies	Page 6, 7 Section 2.6, 3.2
	20b	Present results of all statistical syntheses conducted. If meta-analysis was done, present for each the summary estimate and its precision (e.g. confidence/credible interval) and measures of statistical heterogeneity. If comparing groups, describe the direction of the effect	Page 7 Section 3.3
	20c	Present results of all investigations of possible causes of heterogeneity among study results	Page 7 Section 3.3

(Continued)

Table S1: *Continued*

Section and Topic	Item #	Checklist item	Location where item is reported
	20d	Present results of all sensitivity analyses conducted to assess the robustness of the synthesized results	Page 7 Section 3.3
Reporting biases	21	Present assessments of risk of bias due to missing results (arising from reporting biases) for each synthesis assessed	Page 6 Section 2.6
Certainty of evidence	22	Present assessments of certainty (or confidence) in the body of evidence for each outcome assessed	Page 7 Section 3.3
**DISCUSSION**			
Discussion	23a	Provide a general interpretation of the results in the context of other evidence	Page 12
	23b	Discuss any limitations of the evidence included in the review	Page 12
	23c	Discuss any limitations of the review processes used	Not applicable
	23d	Discuss implications of the results for practice, policy, and future research	Page 16
**OTHER INFORMATION**			
Registration and protocol	24a	Provide registration information for the review, including register name and registration number, or state that the review was not registered	Page 4 Section 2.1
	24b	Indicate where the review protocol can be accessed, or state that a protocol was not prepared	Page 4 Section 2.1
	24c	Describe and explain any amendments to information provided at registration or in the protocol	Page 4 Section 2.1
Support	25	Describe sources of financial or non-financial support for the review, and the role of the funders or sponsors in the review	Page 17
Competing interests	26	Declare any competing interests of review authors	Page 17
Availability of data, code and other materials	27	Report which of the following are publicly available and where they can be found: template data collection forms; data extracted from included studies; data used for all analyses; analytic code; any other materials used in the review	Page 17

From: Page MJ, McKenzie JE, Bossuyt PM, Boutron I, Hoffmann TC, Mulrow CD, et al. The PRISMA 2020 statement: an updated guideline for reporting systematic reviews. BMJ 2021;372:n71. doi: 10.1136/bmj.n71 For more information, visit: http://www.prisma-statement.org/.
